# Clinical Outcomes of Single-Piece Zirconia Implants Compared With Tooth-Supported Fixed Dental Prostheses: A Retrospective Study

**DOI:** 10.7759/cureus.99764

**Published:** 2025-12-21

**Authors:** Aditi Kanitkar, Priyanka Jadhav, Ameer Akhil Ahmed Shaik, Rahul VC Tiwari, Afshan Qureshi, Kapil Laddha, Heena Dixit, Seema Gupta

**Affiliations:** 1 Prosthodontics, Bharati Vidyapeeth (Deemed to be University) Dental College and Hospital, Sangli, IND; 2 Dentistry, Narayana Dental College and Hospital, Nellore, IND; 3 Oral and Maxillofacial Surgery, RKDF Dental College and Research Center, Bhopal, IND; 4 Oral and Maxillofacial Surgery, Vyas Dental College and Hospital, Jodhpur, IND; 5 Dentistry, Monarch Dental, Euless, USA; 6 Public Health, Commisionerate of Health and Family Welfare, Hyderabad, IND; 7 Orthodontics, Kothiwal Dental College and Research Centre, Moradabad, IND

**Keywords:** complications, conventional, dental implants, fixed partial denture, zirconia

## Abstract

Introduction: Replacement of a single missing tooth with evolving treatment alternatives remains a common clinical challenge. This retrospective cohort study aimed to compare the treatment duration and prosthetic/biological complication rates between single-piece zirconia implants and conventional tooth-supported three-unit fixed dental prostheses (FDPs) over a minimum three-year follow-up.

Materials and methods: This study included 200 patients (100 per group) treated between 2018 and 2022 for a single missing mandibular premolar or molar. The implant group received one-piece zirconia implants with immediate/early restoration, whereas the FDP group received porcelain-fused-to-metal or monolithic zirconia three-unit bridges. The treatment duration, number of visits, and complications were retrieved from clinical records. Complication-free survival was analyzed using Kaplan-Meier curves and Cox regression.

Results: Single-piece implants required significantly longer treatment times (157.1 ± 48.3 vs. 46.6 ± 18.9 days; p<0.001) and more clinical visits (5.2 ± 1.1 vs. 3.0 ± 0.8; p<0.001) than FDPs. Overall, prosthetic complication rates were similar (p=0.689), but complication-free survival was significantly higher in the implant group according to the log-rank test (p=0.028), with 56 (56%) implant patients versus 36 (36%) FDP patients remaining complication-free throughout follow-up. The mean time to the first complication or censoring was longer for implants (680.4 vs. 592.3 days; p = 0.024). Multivariate Cox regression analysis showed that restoration type was not an independent predictor of complication risk (p = 0.346) once complications occurred.

Conclusion: Single-piece zirconia implants, despite requiring a longer treatment duration and more appointments, offered superior mid-term complication-free survival compared with conventional three-unit FDPs for replacing a single mandibular posterior tooth. When the preservation of sound adjacent teeth is prioritized, single-piece zirconia implants represent a biologically favorable option for suitable patients.

## Introduction

Replacement of a single missing tooth remains one of the most frequent clinical decisions in restorative dentistry. For decades, the conventional three-unit fixed dental prosthesis (FDP) supported by natural abutments has been the gold standard treatment, offering predictable esthetics and function [1]. However, the preparation of healthy or minimally restored adjacent teeth, the risk of secondary caries, endodontic complications, and irreversible pulp damage have driven the search for tooth-preserving alternatives [1,2].

The introduction of dental implants has revolutionized single-tooth replacement by eliminating the need to sacrifice the sound tooth structure. Despite excellent long-term outcomes, two-piece implant systems typically require multiple surgical and prosthetic appointments, healing periods of 3-6 months, and additional laboratory phases [1-3]. In recent years, single-piece implants, characterized by a one-piece design integrating the implant body and abutment, have gained popularity, particularly monolithic zirconia implants and certain titanium one-piece designs [4]. These systems enable flapless surgery, immediate or early restoration, and delivery of the definitive prosthesis in as few as one to three visits, thereby markedly shortening the overall treatment duration [4]. Another study reported statistically significant marginal bone loss one year after placement of a one-piece zirconia implant system [5].

Although numerous studies have compared the survival and complication rates of tooth- and implant-supported restorations regarding survival and complication rates [6,7], direct evidence comparing true single-piece implants with conventional three-unit tooth-supported bridges remains limited. The existing literature on single-piece implants is largely restricted to short-term case series or specific immediate-loading protocols, with few investigations systematically documenting the total treatment time or long-term prosthetic complications [4,5].

Therefore, the present clinical study aimed to compare the treatment duration, prosthetic and biological complication rates between single-piece dental implants and conventional tooth-supported three-unit FDPs for the replacement of a single missing tooth. The specific objectives were to: (1) compare the total treatment duration, including the number of clinical visits, between single-piece zirconia implants and tooth-supported three-unit FDPs; (2) evaluate and compare the incidence of prosthetic and biological complications over a minimum three-year follow-up; and (3) assess complication-free survival between the two treatment modalities using time-to-event analysis.

## Materials and methods

Study design and location

This retrospective cohort study was conducted at the Department of Oral and Maxillofacial Surgery, RKDF Dental College and Research Center, Bhopal, Madhya Pradesh, India. All clinical data were retrieved from the electronic and paper records of patients treated between January 2015 and August 2020, ensuring a minimum follow-up of three years for every included case.

Ethical considerations

The study protocol was approved by the Institutional Ethics Committee (approval number: RKDF/DC/PG/2021/18E), in accordance with the Declaration of Helsinki (2013). As the study was entirely retrospective and used only anonymized data from routine clinical records, the requirement for individual informed patient consent was waived by the ethics committee. All patient records were de-identified and assigned unique study codes before the analysis to ensure confidentiality.

Sample size estimation

The sample size was determined using the G*Power software (version 3.1.9.2; Heinrich Heine University, Düsseldorf, Germany). The sample size was estimated based on the survival rate of implant-supported single prosthesis (93.3%) and FDP (80%) studied by Alhammadi et al. [8]. A minimum sample size of 200 patients (100 per group) was required to achieve 80% power and a 5% alpha error for the present study.

Eligibility criteria

Patients aged 18-80 years who received treatment for a single missing tooth (premolar or molar) were included. The single-piece implant group comprised of patients restored with one-piece zirconia implants (Z-Systems Z-Look3, Z-Systems AG, Oensingen, Switzerland). The three-unit FDP group comprised patients restored with conventional tooth-supported three-unit FDPs fabricated in either porcelain-fused-to-metal (PFM) or full-contour/monolithic zirconia (IPS e.max ZirCAD, Ivoclar Vivadent AG, Schaan, Liechtenstein). The exclusion criteria were as follows: uncontrolled systemic disease, heavy smoking (>10 cigarettes/day), documented bruxism, periodontal disease with probing depths >5 mm, cantilever or resin-bonded bridges, implant-supported FDPs, incomplete clinical records, or follow-up shorter than 36 months.

Treatment procedures

**Figure 1:**
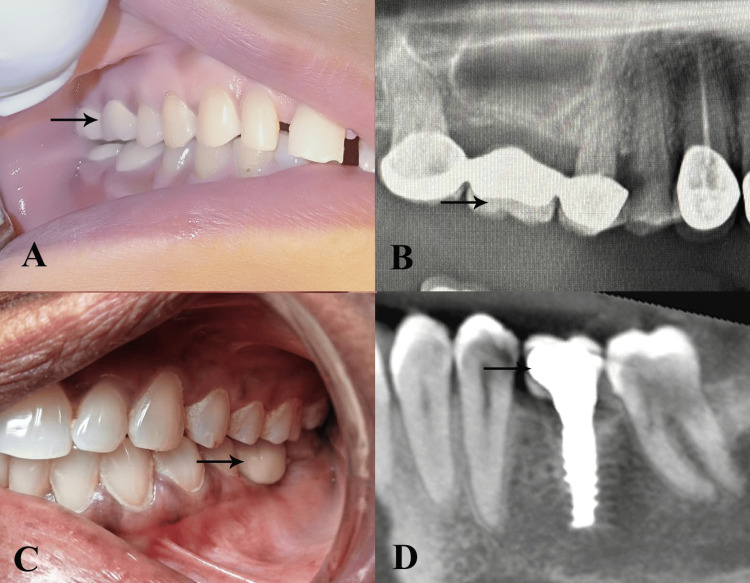
Comparison of tooth-supported three-unit fixed dental prosthesis and single-piece dental implant restoration (A) Intraoral clinical photograph showing a three-unit fixed dental prosthesis replacing a missing right maxillary first molar. The arrow indicates the pontic (false tooth) of the bridge supported by crowned abutment teeth. (B) Cone-beam computed tomography (CBCT) sagittal view of the single-piece dental implant of the mandibular left first molar. The arrow highlights the implant body surrounded by peri-implant bone, demonstrating the absence of a pontic and direct load transfer to the alveolar bone. Original intraoral images of the patients from the study, used with patients' permission.

In the three-unit FDP group (n = 100), abutment teeth were prepared with chamfer margins and final impressions were obtained using polyvinyl siloxane. Temporary acrylic bridges were used during the laboratory phase. Definitive restorations (PFM or monolithic zirconia) were cemented with resin-modified glass ionomer cement (RelyX Luting Plus; 3M Oral Care, St. Paul, MN, USA) after clinical trial and occlusal adjustment (Figure 1A, 1B).

In the single-piece implant group (n = 100), single-piece implants were placed under local anesthesia following the manufacturer’s flapless or minimal-flap protocol. Site preparation was guided by preoperative cone-beam computed tomography (CBCT), and the insertion torque ranged from 35 to 45 N-cm. Immediate provisionalization was performed within 48 hours, and definitive restorations such as lithium disilicate (IPS e.max) or zirconia were delivered 7-21 days after surgery (Figure 1C, 1D).

Data collection and outcome evaluation

Two calibrated examiners independently reviewed all the clinical charts, photographs, and radiographs. Treatment duration was recorded as (1) the total number of clinical appointments from the initial consultation to definitive prosthesis delivery and (2) calendar days between the first and final appointments. Prosthetic and biological complications occurring within the first three years after definitive restoration delivery were classified as complete failure (loss of restoration requiring replacement), major complications (fractures requiring remake or endodontic treatment of abutments), or minor complications (chip, recurrent caries, or loss of retention requiring repair or re-cementation). All events were dated to allow for time-to-event analysis.

Evaluation period

Every included restoration was followed up for a minimum of 36 months after definitive prosthesis delivery. Complications were recorded during this time interval. Patients lost to follow-up before 36 months of age were excluded from the final analysis.

Statistical analysis

Data were analyzed using the Statistical Package for Social Sciences (SPSS) version 23.0 (IBM Corp., Armonk, New York, USA). Descriptive statistics were reported as means ± standard deviations (with 95% Confidence intervals as CIs) for continuous variables and as counts (percentages) for categorical variables. Categorical variables were analyzed with the chi-square test, and continuous variables were compared using an independent samples t-test (due to normal distribution of data confirmed by the Shapiro-Wilk test). The complication-free survival of single-piece implants and three-unit FDPs over three years was compared using Kaplan-Meier survival curves. The log-rank test was used for univariate comparisons between groups. A multivariate Cox proportional hazards regression model was employed to adjust for potential confounders. Statistical significance was set at P < 0.05.

## Results

Based on descriptive analysis, the study groups were well matched for sex (p = 1.000) and showed no significant difference in smoking status (p = 0.102). The incidence of prosthetic complications was comparable between single-piece implants and three-unit FDPs (p = 0.689). However, a notable difference was observed in the overall complication-free survival. A significantly higher proportion of patients with single-piece implants remained complication-free throughout the study than those with three-unit FDPs (p = 0.041). This initial analysis suggests that single implants may offer a superior overall survival profile, warranting further investigation via survival analysis (Table 1).

**Table 1:** Distribution of demographic and clinical characteristics in patients treated with single-piece implants versus three-unit fixed dental prostheses (FDPs). Values are presented as frequency (n) and percentage (%), *p < 0.05 denotes statistical significance using chi-square test.

Parameters	Variables	Single-piece implants (n = 100)		Three-unit FDPs (n = 100)		Chi-square stats	p-value
		n	% within Group	n	% within Group		
Sex	Male	48	48	48	48	0	1
	Female	52	52	52	52		
Smoking	Non smoker	52	52	68	68	2.67	0.102
	Smoker	48	48	32	32		
Prosthetic complications	None	64	64	62	62	0.75	0.689
	Patients with single complication	22	22	18	18		
	Patients with multiple complications	14	14	20	2		
Biological complications	None	76	76	60	6	3.67	0.161
	Patients with single complication	22	22	32	32		
	Patients with multiple complications	2	02	8	08		
Status	Patients with no complications	56	56	36	36	4.81	0.028*
	Patients with complications	44	44	64	64		

Based on comparative analysis, the two treatment groups were well matched for age (p = 0.432). However, significant differences were identified in the treatment logistics and outcomes. The single-piece implant protocol required a substantially longer treatment time (157.1 vs. 46.6 days, p < 0.001) and a greater number of clinical visits (5.2 vs. 3.0, p < 0.001) than three-unit FDPs. Critically, the mean time to a complication or study censoring was significantly longer in the implant group (680.4 vs. 592.3 days, p=0.028). This indicates that, despite a more complex treatment process, single-piece implants were associated with a superior complication-free survival profile over the observation period (Table 2).

**Table 2:** Comparison of age, treatment duration, number of clinical visits, and follow-up time between the single-piece implant and the three-unit fixed dental prostheses (FDPs) group. Values are presented as mean ± standard deviation, *p < 0.05 denotes statistically significance using independent samples t-test.

Variables	Groups	95% Confidence interval for mean	Mean ± SD	T stats	p-value
Age (years)	Single-piece implants	43.45 - 50.95	47.2 ± 9.1	0.79	0.432
	Three-unit FDPs	41.91 - 49.61	45.76 ± 9.33		
Treatment time (Days)	Single-piece implants	150.04 - 164.12	157.08 ± 17.07	42.97	0.001*
	Three-unit FDPs	43.84 - 49.44	46.64 ± 6.78		
Number of visits	Single-piece implants	4.83 - 5.49	5.16 ± 0.8	14.46	0.001*
	Three-unit FDPs	2.76 - 3.32	3.04 ± 0.68		
Time to Event/Censor (Days)	Single-piece implants	542.95 - 817.77	680.36 ± 332.89	2.34	0.024*
	Three-unit FDPs	430.54 - 754.1	592.32 ± 391.93		

Based on the univariate log-rank analysis presented in Table 3, a statistically significant difference in complication-free survival was observed between the two treatment groups (χ² = 4.81, p = 0.028). The single-piece implant group demonstrated a superior survival profile, with 28 (56%) patients remaining complication-free throughout the study period compared to only 18 (36%) in the three-unit FDP group. This result suggests that single-piece implants are associated with a significantly higher probability of long-term, complication-free function than conventional tooth-supported FDPs for the replacement of a single missing tooth.

**Table 3:** Univariate log-rank comparison of overall complication-free survival between single-piece implants and three-unit fixed dental prostheses (FDPs). Event: occurrence of any prosthetic or biological complication; *p < 0.05 denotes statistical significance.

Groups	Total N	N of Event	N of Censored	% of Censored	Chi-square stats	p-value
Single-piece implants	100	44	56	56	4.81	0.028*
Three-unit FDPs	100	64	36	36		

**Figure 2:**
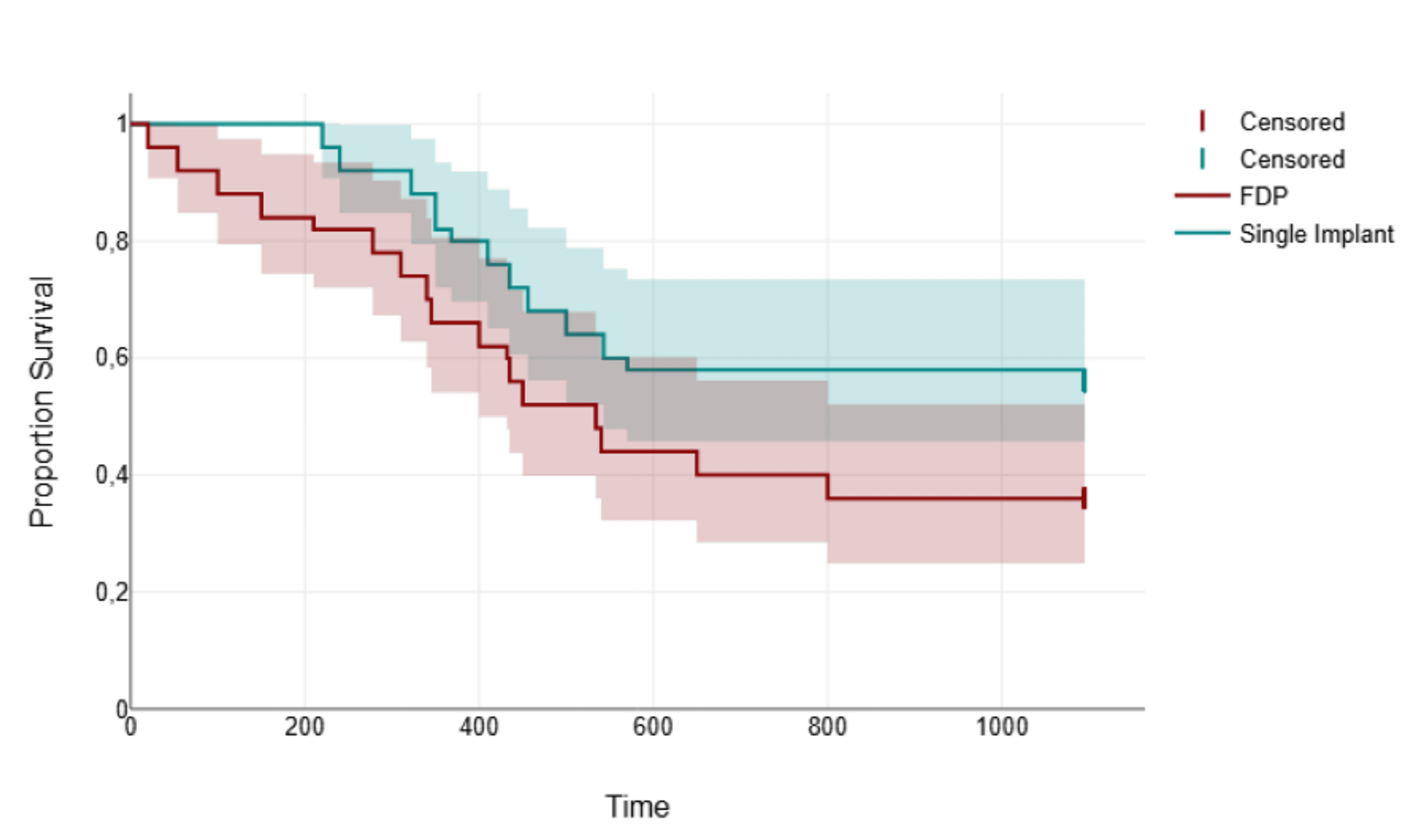
Kaplan-Meier curve showing complication-free survival rates over time for single-piece implants versus three-unit fixed dental prostheses (FDPs). Log-rank test: χ² = 4.81, Event defined as the occurrence of any prosthetic or biological complication. Censored cases indicated by vertical ticks.

Kaplan-Meier analysis showed two distinct lines: one for the single-piece implant group and the other for the three-unit FDP group. The single-piece implant curve was positioned above the three-unit FDP curve, indicating that a higher proportion of patients remained complication-free at any given time point. Vertical drops in the three-unit FDP curve occurred earlier and more steeply, reflecting a higher and earlier incidence of complications in this group (Figure 2).

The multivariate Cox regression model, adjusted for treatment modality and key covariates, revealed that the type of restoration (Three-unit FDP vs. single-piece implant) was not an independent predictor of complication risk (p = 0.346). Similarly, smoking status, treatment time, and the number of visits were not significantly associated. The model identified the occurrence of complications as the primary driver of survival outcomes. Specifically, prosthetic and biological complications are potent independent risk factors for restoration failure. The risk of complications increases dramatically with the number of events, with multiple biological complications conferring a 24-fold greater risk. This indicates that while the initial treatment choice may influence complication rates, as seen in univariate analysis, the actual survival outcome is predominantly determined by the occurrence and, most critically, the accumulation of post-treatment complications, regardless of the prosthesis type (Table 4).

**Table 4:** Multivariate Cox proportional hazards regression analysis of factors associated with complication-free survival. Reference categories: Group: Single-piece implants; Smoking: No; Prosthetic complications: None; Biological complications: None, HR = Hazard Ratio, CI = Confidence Interval, *p < 0.05 denotes statistically significance.

Variables	Coefficients	Hazard ratio	Standard Error	Lower 95% CI	Upper 95% CI	Z stats	p-value
Group (Three-unit FDP)	1.59	4.89	1.68	-1.71	4.89	0.94	0.346
Smoking (Yes)	-0.43	0.65	0.36	-1.14	0.27	1.21	0.227
Treatment time (Days)	0.00	1	0.01	-0.03	0.03	0.02	0.98
Number of visits	0.34	1.41	0.20	-0.04	0.73	1.75	0.081
Prosthetic complications (Single)	1.88	6.58	0.45	1.01	2.76	4.22	0.001*
Prosthetic complications (Multiple)	2.48	11.9	0.42	1.64	3.31	5.83	0.001*
Biological complications (Single)	0.82	2.26	0.35	0.13	1.51	2.32	0.02*
Biological complications (Multiple)	3.20	24.5	0.59	2.04	4.36	5.41	0.001*

**Figure 3:**
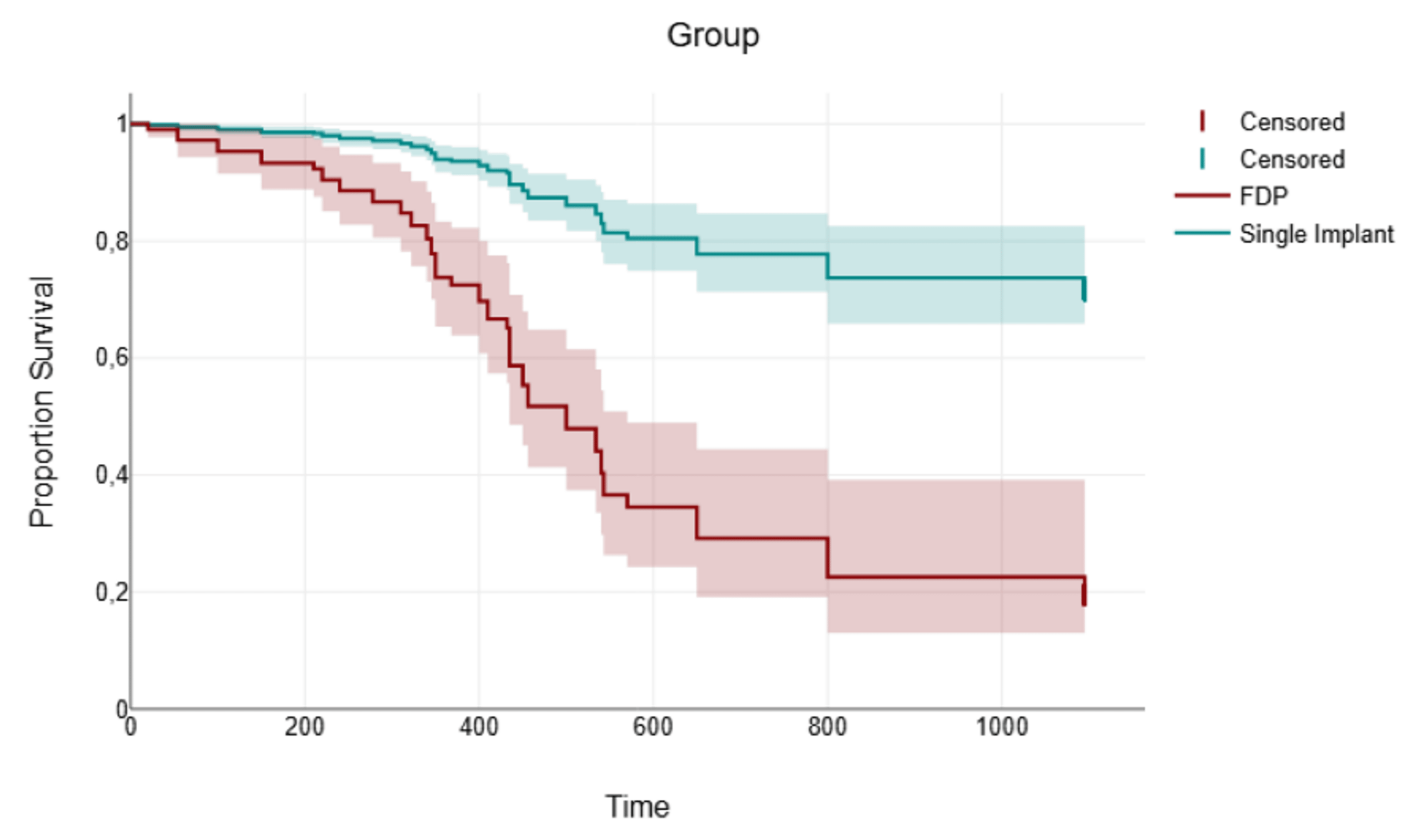
Kaplan-Meier survival curves stratified by type and number of complications in the overall study population. Survival refers to time to first complication (prosthetic or biological); Curves show worse survival with increasing number and severity of complications.

The curve for the single-piece implant group initiated at a higher survival probability and demonstrated a more gradual decline over time, remaining consistently above the three-unit FDP curve. In contrast, the FDP group started at the same point but experienced a steeper and more pronounced drop in survival probability, particularly in the earlier phase of the observation period. This visual separation indicates that patients with single-piece implants experienced a lower risk of encountering complications compared to those with three-unit FDPs (Figure 3).

## Discussion

The present retrospective cohort study compared single-piece zirconia implants with conventional tooth-supported three-unit FDPs to replace a single missing mandibular premolar or molar over a minimum three-year follow-up period. The principal findings revealed a contrasting profile: although single-piece implants required significantly longer treatment duration and more clinical visits, they demonstrated superior complication-free survival compared to three-unit FDPs in univariate analysis. However, when adjusted for confounders in the multivariate Cox regression, the type of restoration was no longer an independent predictor of complication risk. These apparently contradictory results are consistent with emerging evidence in implant and prosthetic dentistry, and highlight the nuanced interplay between treatment complexity, biological response, and long-term maintenance.

The longer treatment time and higher number of appointments observed with single-piece zirconia implants reflect the inherent requirements of contemporary implant therapy, even in “immediate” or “early” loading protocols. Despite the one-piece design and flapless approach intended to simplify workflow, osseointegration monitoring, soft-tissue maturation around transmucosal zirconia, and definitive prosthetic delivery still necessitate additional visits in routine private-practice settings [4]. Kohal et al. [5] in a prospective study of one-piece zirconia implants, reported 90.8% survival rate after three years and mean marginal bone loss of 1.45 m. However, univariate analysis did not confirm any significant parameter related to marginal bone loss. The present study reported complications in 44 patients (44%) after three years. Kohal et al. [9] reported that single-piece zirconia implants showed lower survival rates owing to peri-implantitis. Bragger et al. [10] reported more clinical visits for single-crown implants compared to conventional three-unit FDPs for single-tooth replacement; however, they required higher laboratory costs for FDP than implants. A previous study reported that single implants are more cost-effective than conventional three-unit FDPs [11].

Despite the prolonged treatment phase, single-piece implants exhibited fewer overall complications and delayed time-to-first events in the univariate analysis. This advantage likely stems from the elimination of abutment tooth preparation and microgap-associated risks inherent to tooth-supported FDPs. The preparation of vital or minimally restored abutments carries well-documented risks of pulp necrosis (3-15% at 10 years) and secondary caries at crown margins [12]. In the current cohort, biological complications in the FDP group were dominated by endodontic issues and decementation, consistent with large registry data showing a cumulative 5-year survival rate of 81.8%, loss of pulp vitality (32%), caries of abutment teeth (9.1%), loss of retention (16.1%), and material fractures (5.9%) [13]. Conversely, one-piece zirconia implants avoid the implant-abutment interface entirely, eliminating the major pathway for peri-implantitis initiation observed in two-piece systems [14]. Balmer et al. [15] reported a higher survival rate with less marginal loss with single-piece dental implants than with a three-unit FDP.

The loss of statistical significance in the multivariate analysis indicates that once a complication occurs, the trajectory toward further events or failure is largely independent of the original restoration type. This observation aligns with the concept of “clustering of complications,” whereby an initial biological insult (such as peri-implant mucositis or marginal caries) predisposes the site to subsequent events regardless of whether the restoration is implant- or tooth-supported [16]. The 24-fold increase in hazard associated with multiple biological complications in the Cox model underscores the critical importance of meticulous maintenance protocols in both modalities.

Clinical implications

For patients and clinicians prioritizing speed and minimal appointments, conventional three-unit FDPs remain a viable option, particularly when adjacent teeth already present with restorations requiring crowns. However, when preservation of sound abutment structure is a primary goal and patients accept a longer initial treatment duration, single-piece zirconia implants offer a more predictable long-term complication-free outcome, especially in periodontally healthy, non-bruxing individuals. The superior univariate survival of implants supports the expansion of their indications beyond immediate loading scenarios into routine single-tooth replacement in the posterior mandible.

Limitations

This retrospective design introduces potential selection and information bias. The study was limited to mandibular premolars/molars restored with one specific zirconia implant system and two FDP materials; the results may not be generalizable to maxillary teeth, two-piece implants, or metal-ceramic implant crowns. Follow-up beyond seven years is still needed to confirm whether the early advantage of single-piece implants persists in the long term. Finally, patient-centered outcomes (satisfaction, cost-effectiveness, and chewing efficiency) were not assessed.

## Conclusions

Single-piece zirconia implants and conventional three-unit FDPs both provided reliable restoration of a single missing mandibular tooth over a minimum three-year follow-up. Although implants required a longer treatment duration and more clinical visits, they demonstrated a more favorable mid-term complication-free profile in univariate analysis. However, because this study is retrospective and subject to potential selection bias and unmeasured confounding, these findings should be interpreted as associative rather than definitive, and not as proof of biological superiority.

The multivariate Cox regression analysis further revealed that the occurrence and accumulation of biological complications, rather than the type of restoration itself, is the strongest determinant of long-term failure risk. This underscores the importance of meticulous maintenance protocols for both implants and FDPs. When adjacent teeth are intact, and patients accept a longer treatment process, single-piece zirconia implants represent a conservative and potentially advantageous option; however, well-designed prospective studies are needed to confirm their long-term biological and prosthetic performance.
